# Novel Approaches and Cognitive Neuroscience Perspectives on False Memory and Deception

**DOI:** 10.3389/fpsyg.2022.721961

**Published:** 2022-03-21

**Authors:** Michael P. Toglia, Joseph Schmuller, Britni G. Surprenant, Katherine C. Hooper, Natasha N. DeMeo, Brett L. Wallace

**Affiliations:** ^1^Department of Psychology, Cornell University, Ithaca, NY, United States; ^2^Department of Psychology, University of North Florida, Jacksonville, FL, United States; ^3^Department of Psychology, University of Georgia, Athens, GA, United States; ^4^Department of Biobehavioral Health, The Pennsylvania State University, University Park, PA, United States; ^5^School of Psychology, Florida Institute of Technology, Melbourne, FL, United States

**Keywords:** DRM paradigm, lexical decision task, fNIRS, deception, fuzzy trace theory, activation-monitoring theory, spreading activation, cognitive neuroscience

## Abstract

The DRM (Deese–Roediger–McDermott) paradigm produces robust false memories of non-presented critical words. After studying a thematic word list (e.g., *bed, rest*, and *pillow*) participants falsely remember the critical item “sleep.” We report two false memory experiments. Study One introduces a novel use of the lexical decision task (LDT) to prime critical words. Participants see two letter-strings and make timed responses indicating whether they are both words. The word pairs Night-Bed and Dream-Thweeb both prime “sleep” but only one pair contains two words. Our primary purpose is to introduce this new methodology *via* two pilot experiments. The results, considered preliminary, are promising as they indicate that participants were as likely to recognize critical words (false memories) and presented words (true memories) just as when studying thematic lists. Study Two actually employs the standard DRM lists so that semantic priming is in play there as well. The second study, however, uses functional near-infrared spectroscopy (fNIRS) to measure activity in the prefrontal cortex during a DRM task which includes a deception phase where participants intentionally lie about critical lures. False and true memories occurred at high levels and activated many of the same brain regions but, compared to true memories, cortical activity was higher for false memories and lies. Accuracy findings are accompanied by confidence and reaction time results. Both investigations suggest that it is difficult to distinguish accurate from inaccurate memories. We explain results in terms of activation-monitoring theory and Fuzzy Trace Theory. We provide real world implications and suggest extending the present research to varying age groups and special populations. A nagging question has not been satisfactorily answered: Could neural pathways exist that signal the presence of false memories and lies? Answering this question will require imaging experiments that focus on regions of distinction such as the anterior prefrontal cortex.

## Introduction and Historical Backdrop

It is well known that accurate remembering may fall under the influence of errors of omission and/or of commission. Nevertheless, memory generally serves us quite well, even with gaps in recounting an event. Gap-related failures due to errors of omission are often attributed to forgetting, a natural feature of cognitive functioning.

Our focus, however, is on errors of commission that typify faulty remembrances. These flaws in remembering have come to be known as *false memories*. They occur when we remember events in a distorted manner or come to have memories for events that never happened. The term “false memory” appeared in the early 1990s in the eyewitness memory literature (Loftus, 1993) and with Peter and Pamela Freyd’s founding of the False Memory Syndrome Foundation in 1992. The term’s acceptance solidified shortly after the publication of the seminal article by Roediger and McDermott (1995). While “false memory” has become an umbrella encompassing many forms of deficits and alterations of human memory, the psychological literature addressing the frailties of memory stretches back well over a 100 years (Ebbinghaus, 1885; Munsterberg, 1908). Explanations for how and why false memories occur emphasize the role of reconstructive processes in retrieval (see Bartlett, 1932 for cogent, early arguments). Roediger and McDermott (1995) went a step further: They made the case that all remembering, accurate or not, depends on reconstruction.

Inaccurate memories share a common trait in that they contain at least slivers of truth. Even our strongest true memories, however, have false components – as is the case in flashbulb memories (Hornstein et al., 2003), This overlapping characteristic is consistent with the notion that illusory memories, though errant, maintain some aspects of real experiences, often sharing central features of meaning as in recalling “hurt on a school playground” rather than “hurt in a city park” (see Brainerd et al., 2008 for similar arguments).

Many factors contribute to the difficulty in distinguishing true recollections from false ones (Marche et al., 2010). Further blurring the distinction is that illusory memories are highly believed, often at levels comparable to true memories (Toglia et al., 1999; Shaw, 2020).

The concept of belief raises another form of factual inaccuracy – the deliberate inaccuracy when someone knowingly lies. False memories in most instances are not, and should not, be equated with lies (Ceci et al., 1987; Loftus, 2005). Yet some similarities are evident: Liars bolster believability by inserting some degree of truth into an overall false narrative or by embedding false information in a generally true narrative. In fact, these “half-truths”, if believed, might serve as a lynchpin for the creation of a false memory (Otgaar and Baker, 2018). Empirical and theoretical studies of accuracy, inaccuracy, and deception, across many subfields of psychology as well as forensic science and neuroscience, are important because false memories and lies are common in daily life. Regarding the latter, intent is involved in fibs, white lies, and whoppers. False memories are not considered intentional (but see our Study 2 reported later). They take shape from misinformation or from misremembering. Specifically, some real-life illusory recollections form from a blend of unintentional or purposeful misinformation, and/or from schematic knowledge represented in conceptual networks in semantic memory. These inaccurate additions to long-term declarative memory are pervasive in real-world settings. Significantly, research shows false memories to be long lasting under experimental conditions (Toglia et al., 1999; Seamon et al., 2002; Brainerd and Reyna, 2005).

### The Deese–Roediger–McDermott Illusion

The DRM paradigm (Deese, 1959; Roediger and McDermott, 1995) involves standard verbal learning of lists of semantically related words. This paradigm and variants have been the foundation of thousands of false memory experiments, including the studies we report in this article. Kirkpatrick (1894) conducted and reported a DRM-like experiment generally regarded as the first false memory study. Her participants studied several groups of verbal items, each containing thematic connections. Their recall protocols revealed intrusions of words associated with the study lists. In her discussion section she wrote:

There were some incidental cases of false recall. About a week before… I had said ten words to the students. Many of these were evoked and placed on the lists as if they were part of it. Again, it seems that when words such as “roll,” “thimble,” and “knife” were pronounced, many students thought of “thread,” “needle,” and “fork,” which are so often associated with them. The result was that many of these words were evoked as belonging to the list. This is an excellent illustration of how things suggested to a person during an experience can be honestly reported by that person as part of that experience. (pp. 608, 609).

Note that Kirkpatrick mentions “incidental cases of false recall” and that suggestions during an experience “can be honestly reported by that person as part of that experience.” These quotes comport with our earlier comments that false recollections are not viewed as intentional nor considered lies.

One hundred years after Kirkpatrick’s study, Roediger and McDermott (1995) revived Deese’s (1959) associative memory procedure used effectively, primarily in the laboratory, for more than 25 years, to investigate an array of false memory phenomena. In a typical DRM study participants study lists of words. Each list consists of semantically related items, some more than others, that converge upon a critical item (often referred to as a “critical lure”) that does not appear during study. For instance, consider *chair* as the critical missing item for this list*: table, sit, legs, couch, desk, recliner, sofa, wood, cushion, swivel, stool, sitting, rocking, and bench.* List presentation sometimes precedes a brief delay to buffer the effects of short-term memory. Then participants have to recall or recognize as many of the presented words as they can and in doing so, they often “remember” the critical item as part of the study list. False recall usually occurs 50% of the time, while false recognition of critical items occurs at rates that approach 80%. These false memory effects are sometimes referred to in the literature as the “DRM illusion.” Thus, illusory memories are very compelling, so much so that their rate of occurrence very often exceeds levels of accurate (true) memory (Toglia et al., 1999; Brainerd and Reyna, 2005; Bui et al., 2013; Pimentel and Albuquerque, 2013).

Beyond their frequency, other findings speak to the phenomenological experience of false memories. For example, participants typically express very high confidence in memories that are inaccurate (Roediger and McDermott, 1995). In addition to measuring belief in their recall or recognition of words, in some experiments, participants categorize their memories with either a “remember” judgment (a detailed, vivid independent recollection), a “know” judgment (a general sense of familiarity without any independent recollection), or a “guess” judgment. Participants often claim they “remember” critical lures as frequently as the studied words. Thus, false memories are very robust. This alarming conclusion prompted researchers to examine ways to potentially diminish the creation and occurrence of false memories.

Experimental approaches to reduce the DRM illusion include warning participants about the thematic nature of the lists to be studied by providing them with sample lists and their critical lures. These explicit examples are given to make the point that the experimenter is trying to trick participants to remember non-presented words and to urge them not to fall into this trap. These “warning” studies report mixed results, yet generally show warnings often fail to reduce the DRM illusion (Neuschatz et al., 2001).

### Why the Deese–Roediger–McDermott Paradigm

The DRM approach to the study of false memory has dominated this literature over the last two decades. An easily conducted experimental procedure, it yields impressive levels of illusory memory which is at the root of this dominance. Arguably the results have wide ranging applications beyond word lists (Brainerd and Reyna, 2002). For purposes of this article, these include implications for false memory in legal contexts. Such implications have met skepticism based on ecological validity. This is a fair point. We ask readers to refrain from judging the degree of external validity until we have advanced our concluding remarks.

For the moment, consider that forensic interviewers might introduce thematically related words and phrases weaved throughout the questioning. The related material could converge on topics that can lead an interviewee to provide false memories as answers. Similarly, police frequently use aggressive methods (the Reid Technique), combined with establishing an accusatory atmosphere when interrogating suspects who they presume guilty (Kassin and Kiechel, 1996; Kassin and Gudjonsson, 2004).

When the investigating detective’s goal is to force a confession, the detective might make repeated accusations of guilt, infuse misleading information and lies, and discuss criminal themes. In the most extreme form of forced confessions known as internalized false confessions (Kassin and Wrightsman, 1985; Kassin, 2008) the innocent suspect comes to believe in his or her guilt substantiated by new, confabulated, memories that are false.

While this chapter focuses on falsely recalled and/or recognized words, in terms of generalizability the literature is replete with DRM-consistent memory results observed across a wide range of stimulus materials. These include memory for pictures (Israel and Schacter, 1997; Kouststaal et al., 2003; Foley and Foy, 2008), memory for sentences (Bransford and Franks, 1971; Bransford et al., 1972; Matzen and Benjamin, 2013), memory for text (Bower et al., 1979; Reyna and Kiernan, 1994), remembering pragmatic inferences (Brewer, 1977; Barclay et al., 1984), and remembering scenes (Dechterenko et al., 2021). Consistent results also occur in naturalistic contexts, as in memory for the contents of a graduate student’s office (Brewer and Treyens, 1981) and memory for a professor’s lecture and behaviors during its delivery (Neuschatz et al., 2002). Accompanying these many forms of behavioral data establishing the pervasive occurrence of false memories, is an ever-increasing literature that addresses the neural correlates of false memory. Expansive meta-analyses of dozens of fMRI studies have implicated several regions of the frontal, temporal, and parietal cortices in the encoding and retrieval phases of false memory, with the superior prefrontal gyrus being most significant (Kurkela and Dennis, 2016; Yu et al., 2019).

These materials and contexts, as well as behavioral and neurophysiological investigations, clearly involve a variety of paradigms that all rely on semantic bases in fomenting false memories. The semantic/thematic nature of experimental materials, even though they may vary widely, permit theories to account for most effects across these stimulus materials.

### Theoretical Explanations of False Memory Effects

Any adequate theory of memory must account for both true and false memories. We now outline two theories that satisfy this requirement.

We begin with Activation-Monitoring Theory (AMT), a dual-process theory. Here the processes are cognitive operations, activation and monitoring. Activation primarily happens at encoding. Monitoring is largely a retrieval process, though both can operate at encoding and retrieval (Roediger et al., 2001). The term “activation” is from spreading activation theory which represents concepts and their properties in associative networks (e.g., Collins and Loftus, 1975). Items in long-term memory are interconnected nodes throughout the network, an extensive web-like structure. Connections between two concepts vary in their “semantic distance,” determined by the strength of the connection. Strongly related terms (e.g., chair-table) are separated by short semantic distances, while weaker-bonded items (e.g., chair-bench) are linked at longer distances. We use here the examples from the DRM list mentioned earlier to facilitate the discussion of activation as it applies to an encoding phase in a false memory experiment. According to AMT, as the participant hears a list, he or she encodes each word into a network node and activation spreads to connected words; stronger connections enjoy greater activation. Many of the presented words receive substantial activation, repeatedly so, and this supports true memory. Critical lures, however, also receive repeated activation from the semantically-related, presented list items. At retrieval participants engage in the second activity of the dual processes, monitoring, which involves editing of whether or not to commit to recalling or recognizing a word. Highly activated items, the presented words and critical lures, are likely to cross the decision threshold (i.e., the “criterion” in recognition memory models) for output at test.

Another approach to understanding the DRM illusion is Fuzzy Trace Theory (FTT) advanced by Reyna and Brainerd (1995). They proposed that stimuli are encoded in two independent ways. FTT is thus a dual-process theory. Unlike AMT, its processes target the development, independently, of two types of memory traces. One involves verbatim encoding, producing a trace of exact, detailed information as in the list words. The other involves coding for gist, creating a trace of general characteristics, like the list words relating to a theme (e.g., chair). Both verbatim and gist representations contribute to high levels of true memory. For illusory memory, the focus is on gist processing. Participants frequently falsely recall/recognize “chair” as a list item. They cannot possibly access a verbatim trace of “chair” because it was not presented. They can, however, rely on a strong gist trace because “chair” is the best descriptor of the theme of the studied list.

We now present two pilot studies, collectively referred to as Study 1. We begin with a lexical decision task investigation, involving a student project and a follow-up experiment, the second author designed with spreading activation/AMT in mind.

## Study 1

### Introduction

Introduced by Meyer and Schvaneveldt (1971), the lexical decision task (LDT) has seen wide usage in the study of semantic priming processes in neurotypical populations of all ages (see Plaut and Booth, 2000 for theoretical arguments), in individuals with linguistic deficits (e.g., aphasia, Miberg and Blumstein, 1981; dyslexia, Martens and de Jong, 2006), and with memory impairments (e.g., Alzheimer’s disease, Ober and Shenaut, 1988). In one version of Meyer and Schvaneveldt’s (1971) procedure, the participant sees two letter-strings on each trial, one above the other. The task is to press one button if both letter-strings are words, and another if at least one-letter string is not a word. The two strings remain visible until the response, and the dependent variable is the time between the beginning of the trial and the participant’s response. One typical result is that participants correctly respond faster when the two letter-strings are semantically related words than when they are semantically unrelated words. This is consistent with spreading activation theory (Collins and Loftus, 1975).

**FIGURE 1:**
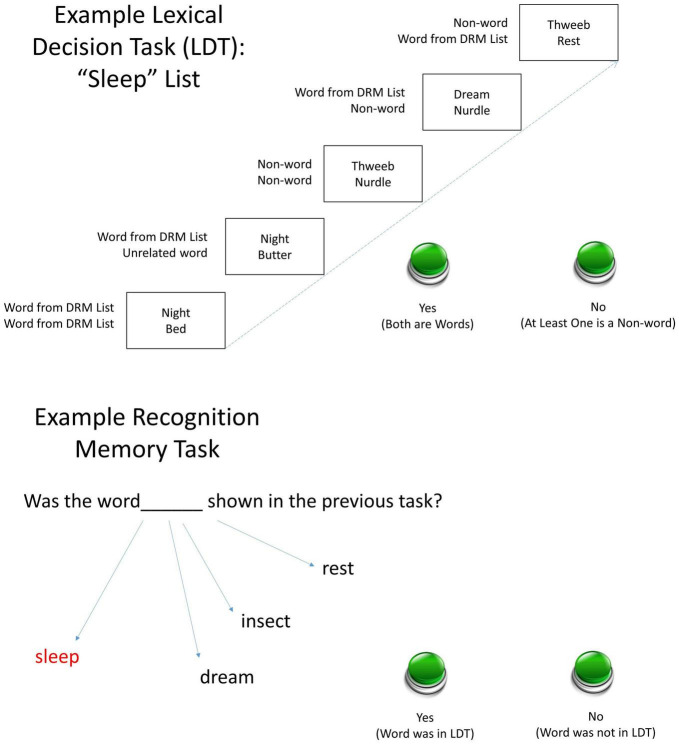
Examples of the two-item trial types during the LDT phase **(Top)** and the recognition memory test **(Lower)**. Error bars show standard errors. Participants completed the LDT encoding phase involving two-item letter strings composed of words from six DRM lists and/or non-words, followed by a recognition testing phase. Accuracy and reaction time (RT) were recorded.

We used this two-word procedure to fully leverage the potent priming effects of DRM list words. The DRM procedure in this study appears in a novel way: Instead of using the LDT in its standard method as an assessment for false recognition, the LDT was the semantic priming stimulus for subsequent false recognition. The words in the LDT came from DRM lists. A recognition test followed each LDT block. The test contained the critical lures from the DRM word lists primed in the preceding LDT trials. For example, an LDT trial could present two words from the same DRM list (e.g., “night” and “bed” from the *sleep* list), two words from different DRM lists (“night” and “butter” from the *bread* list), a word and a non-word, or two non-words. Figure 1 shows examples.

Our long-term goal is to understand *behavioral* and *neurophysiological* underpinnings of false recognition by utilizing this novel *behavioral* approach as well as a *neurophysiological* approach that leverages functional near-infrared spectroscopy (fNIRS, described in Study 2) concurrent with the LDT trials and recognition trials.

The present report only addresses a short-term objective achieved by implementing the *behavioral* approach in a classroom experiment and in a small follow-up experiment. In doing so we accomplish the overarching objective of introducing this new and novel paradigm, consistent with the purpose of the volume in which the present article appears. We predicted that semantic priming stemming from LDT trials would successfully produce false memories on a subsequent old/new recognition test.

At this juncture, we have not gathered data for the *neurophysiological* approach, which will involve an already-designed separate fNIRS study. Ultimately, this subsequent fNIRS experiment will include an LDT paradigm similar to the behavioral work presented here. Results in the literature that delineate neural correlates of true versus false recognition are incomplete, as we will describe when we present Study 2. Therefore, when we launch an fNIRS experiment involving LDT we will focus on determining cortical activation patterns that reflect similarities and differences between true and false recognition when they are based on priming that occurs on LDT trials. What might we find in an LDT investigation that includes an examination of neural correlates? We can glean clues from the brain imaging experiment we report later. In this second experiment we used a relatively new brain imaging technique, functional near-infrared spectroscopy fNIRS). We measured activity in the prefrontal cortex in an attempt to dissociate false recognition memories from true recognition.

### Method

#### Student Project

In an initial evaluation of the LDT-priming method, eight college students (five female and three male; mean age = 21.14) participated in three blocks of LDTs with a recognition block after each LDT block. This was part of a classroom project. The procedural details we describe are complemented by Figure 1. Each participant worked at a Dell 780 Optiplex computer equipped with a standard keyboard, monitor, and mouse. Each computer ran under Windows 7. The experiment ran in a program called PEAK which presented the stimuli and recorded the responses and response times. Words for the LDT came from DRM lists.

We randomly chose six categories, referred to by critical lures for six DRM list themes for the classroom experiment (*anger, sleep, cold, girl, thief*, and *chair*), two categories for each LDT block. Each category had a critical lure. Two critical lures appeared in each block of the recognition task.

Each participant sat approximately two feet away from his or her monitor with fingers resting on the “1” and “2” keys of the keyboard. The experiment consisted of six blocks. Blocks one, three, and five presented the lexical decision task, blocks two, four, and six presented the recognition task.

A fixation point appeared in the center of the screen. The participant pressed the spacebar to initiate a trial. On each LDT trial, two letter-strings appeared, one above the fixation point, one below. Each pair consisted of either two related words (W-RW), two unrelated words (W-URW), a word above a non-word (W-NW), a non-word above a word (NW-W), or two non-words (NW-NW). The task was to push the “1”-key if both were words, or the “2”-key otherwise. The dependent variable was the time from the appearance of the pair until the button-push.

On each recognition trial, a word appeared in the center of the screen. Each word either occurred in the preceding LDT block or it did not. Of the words that did not, two were critical lures. The task was to push the “1”-key if the word appeared in the preceding LDT block, or the “2”-key if it did not. The dependent variables measured during the memory test were (a) the time from the appearance of the word until the button-push and (b) accuracy.

Each participant completed three LDT blocks. Each LDT block consisted of 25 trials and preceded a recognition block consisting of 20 trials. Within each block, each stimulus appeared in a different random order for each participant.

#### Follow-Up Experiment

Though testing only six participants (four female and two male; mean age = 21.63), the follow-up experiment to the student laboratory project expanded the scope to include 12 DRM lists across six separate LDT blocks. The follow-up doubled the number priming lists, and differed from the lab study by including new test words not thematically related to the 12 DRM lists and did not measure RT. Thus, the follow-up experiment is not strictly a replication and for this reason we could not pool the data from these two pilot efforts. For each LDT block in the second pilot study, pairs of items were (a) words from the top 12 associates in each of two ordered DRM lists (e.g., “sleep” and “rough” lists), (b) 18 non-words of similar length, and (c) six words from unpresented DRM lists that were unrelated to either presented list.

More specifically, the six unrelated items were from six DRM lists which were not associated with any items on the subsequent recognition test. These formed 24 pairs of items: three DRM word-pairs from each list (e.g., “bed” and “rest” as a pair; six pairs total), three DRM list words from each list paired with non-words (six pairs total), three DRM list words from each list paired with unrelated words (six total), and six non-word pairs. Similar to the classroom study, participants judged whether or not the pair of items were both words, by responding yes or no, respectively.

The pairs resulted in 12 yes items and 12 no items if answered correctly, although correct responding was not recorded for the LDT encoding phase. Incorrect decisions are rare, and even the occurrence of a few mistakes would likely not interfere with exposure to the sources of priming which was the overarching purpose.

Each corresponding recognition block consisted of 24 words (presented individually). The task was to decide if each word appeared in the preceding LDT block, the same procedure when asking for old/new recognition judgments.

For each of the six recognition blocks, we randomly selected six of the 12 associates from each DRM list (“targets”), the critical item from each of the two lists (“critical lures”), two non-presented items from positions 13–15 of each of the two presented lists (“low associates” from the sleep/rough etc. lists), two critical lures from non-presented DRM lists (“pseudo critical lures”), and two low associates from positions 13–15 of each of two non-presented DRM lists (“pseudo low associates”). This resulted in 12 items that previously appeared and 12 that did not. During the recognition block, the dependent variable was accuracy: RT was not part of the follow-up, which we designed primarily to further pilot procedural details, while still allowing general comparisons with the laboratory project.

### Results

**FIGURE 2:**
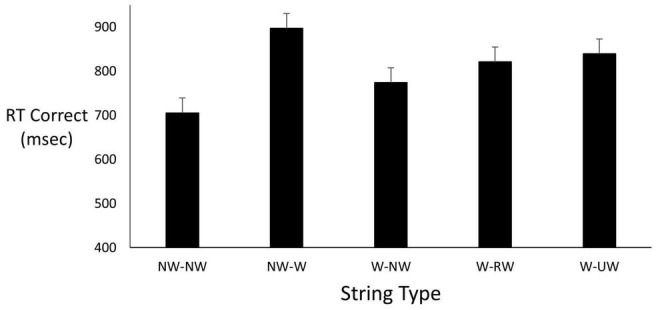
Mean correct RT as a function of string type. String types abbreviated here are identified in the top panel of Figure 1.

Given the small sample sizes in both the project and subsequent experiment, we only report descriptive (means) information which we believe speaks for itself. These are preliminary findings and are to be interpreted with care as we have not fully tested this new paradigm and its efficacy. Again, we are introducing this paradigm into the LDT and false memory literatures. For the student project, Figure 2 shows reaction time values for the five different types of LDT encoding trials. As expected, the NW-NW combination was associated with the fastest times. Turning to testing, results indicated that participants were almost six times as likely to falsely recognize a critical lure than an unrelated lure (0.69 vs. 0.12). Regarding correct recognition of target words, participants were highly accurate with performance near ceiling. Unlike many studies in the literature, correct recognition exceeded false recognition rather than the opposite outcome.

Seven of the eight participants averaged 255 ms faster responses to falsely recognize a critical lure than to falsely recognize an unrelated lure (695 ms vs. 950 ms). Correctly classifying a critical lure took 373 ms longer (1,183 ms) than correctly classifying an unrelated lure (810 ms), which was about the same decision time for correctly responding to target words.

As noted, accuracy was the only dependent variable for the follow-up experiment. The proportion correct recognition for target words was 0.60, while false memory of critical lures occurred at a 0.50 rate. As in the student project, true memory exceeded false memory. Low associates from presented DRM lists were falsely recognized at a proportion of 0.22.

Pseudo test items were either “critical lures” (PCLs) or “low associates” (PLAs) selected from non-presented DRM lists. The PCLs can be equated with unrelated lures in the student study and novel lures in the fNIRS experiment reported in the next section. The proportion of errors, yes responses to PCLs was 0.13, while for PLAs participants chose yes at an errant proportion of 0.27. Critical lure illusory memory, as expected, exceeded all other forms of false recognition.

### Discussion

These studies support, we believe, the idea that the LDT/Recognition paradigm presented here is a unique way of studying both the processes involved in false memory and those involved in lexical decision making. Given that these are pilots, we caution that the implications of the findings are preliminary. Yet the results are promising as our LDT procedure does prime false memory, and the extent of false memory observed is an indicator, we argue, of spreading activation that occurs during the LDT trials. In AMT terms, the LDT priming is sufficiently robust as it thwarts monitoring required to prevent creating significant amounts of illusory memories. This account does not rule out a Fuzzy Trace Theory explanation. Priming based on verbatim processes per FTT produced very good true memory. Gist processes also aided accuracy while instilling themes supporting critical lure and low associate lure false memories. Low associates were falsely recognized far less often than critical words. This finding is predicted by FTT because these associates are much less gist consistent than critical lures that are the best exemplars of the DRM themes. Note that AMT expects this outcome as well because the critical lures are semantically linked to DRM list words more closely than are low associates. These accuracy/inaccuracy arguments are further supported by our response speed findings to which we now turn.

The fastest response times in the student project occurred on trials where both strings were non-words as participants quickly confirm neither item points to an entry in semantic memory. This is a common outcome (Meyer and Schvaneveldt, 1971). Critical lures are likely to be strongly activated due to their close links to target words compared to more semantically distant links with unrelated lures. AMT is consistent with quicker false affirmation responses to critical than to unrelated lures. Similarly, FTT predicts this pattern – during LDT trials durable theme-consistent traces of critical words are developed *via* repeated cueing of gist, while unrelated lures at test are generally gist-inconsistent.

This paradigm adds the dimension of individual differences analysis. Although only a small sample participated in the student laboratory project at least one interesting non-typical pattern emerged. In the Section “Results,” we mentioned for the lab project that 7 of the 8 participants falsely recognized a critical lure faster than an unrelated lure. The eighth participant also showed an atypical result in the LDT, correctly responding faster to W-UW pairs than to W-RW pairs. We note this case, not to speculate on its theoretical significance, but to express a caution. Specifically, rather than search for a one-size-fits-all theory, we should perhaps focus on the particular processes that individuals use. We further mention individual differences in the General Discussion section.

## Study 2

### Introduction

To elucidate the neural distinctions between real (true) memories, false memories, and lies, researchers have used various methods and experimental paradigms. Different but overlapping bilateral regions of the brain have been implicated in each of these processes, with the prefrontal cortex playing a major role in all three.

True memories activate several regions of the prefrontal cortex depending on the type of task and the material being encoded, with greater levels of activation predicting a higher likelihood of retrieval success (for review see Fletcher and Henson, 2001; for meta-analyses see Davachi et al., 2001; Barde and Thompson-Schill, 2002; Wager and Smith, 2003). False memories activate many of the same prefrontal areas that true memories do: This might reflect this region’s involvement in monitoring, verifying, and reporting retrieved information (for meta-analysis see Kurkela and Dennis, 2016). Lying is an effortful and elaborative process that requires integrating information in working memory, inhibiting one’s own behavior, and monitoring others’ behavior, so it is not surprising that it requires even more prefrontal resources than truth-telling and false memories (for meta-analysis see Yu et al., 2019).

For all three processes researchers report that the specific frontal areas activated depends on the methodologies used and task-related factors such as the procedure, the stimuli used, the participants’ goals and intentions, and the social context (Fletcher and Henson, 2001; Barde and Thompson-Schill, 2002; Wager and Smith, 2003; Kurkela and Dennis, 2016; Yu et al., 2019; Pinti et al., 2021). Due to these variations in methodology, as well as the small sample sizes common in brain imaging studies, the specific role of particular frontal areas in memory processes is still unclear.

#### Functional Near-Infrared Spectroscopy

Functional near-infrared spectroscopy is a relatively new brain imaging technique that allows researchers to study memory processes in the cortex. Light emitting diodes (LEDs) placed on the scalp safely emit two wavelengths of infrared light that hemoglobin in the blood absorbs. Detectors measure the light that is refracted and the modified Beer-Lambert law enables us to quantify the level of blood oxygenation. Like the blood-oxygen-level-dependent (BOLD) response in fMRI, blood oxygenation in fNIRS is a measure of cortical activation. fNIRS has the advantage of being inexpensive, portable, not requiring participants to lay prone and still, and not requiring that trials be presented in blocks. Importantly, fNIRS has higher temporal resolution, but lower spatial resolution, than fMRI (Ferrari and Quaresima, 2012). Numerous studies, including those involving true memories, false memories, and deception, have validated fNIRS results by comparing them to those obtained with fMRI (Tian et al., 2009; Ding et al., 2013, 2014; Bhutta et al., 2015; Li et al., 2018; Pinti et al., 2021).

We used fNIRS to measure prefrontal cortical responses in a DRM false memory paradigm. Our experimental design concisely evaluates true memories, false memories, and lying in a single within-participants study. To our knowledge, only one other study, Abe et al. (2008) published more than 10 years ago, has compared all three in a single investigation.

### Method

The sample was 33 college students, 28 females and 5 males, with an average age of 21.79 ± 4.46 years old. A majority of the participants were right-handed (*n* = 30) and white (*n* = 19). Each participant entered the laboratory individually. We used the NIRScout continuous-wave fNIRS system from NIRx Medical Technologies (Berlin, Germany) to measure blood oxygenation levels in the entire prefrontal cortex. We did this with a 38-channel prefrontal montage containing 16 two-wavelength (760 and 850 nm) LED sources and 12 avalanche photodiode detectors set 3 cm apart on the scalp. The step frequency of NIRScout is 62.5 Hz, thus the time-multiplexed sampling rate of each of our channels is 3.91 Hz. To record the raw data we used the NIRx NIRStar acquisition software (version 15.2 NIRx Medical Technologies LLC, Berlin, Germany) which detects refracted light from the sources and calculates oxyhemoglobin levels for each channel in the montage. This allowed us to compare relative levels of brain activity in each prefrontal region.

Inquisit presentation software (Inquisit 5, 2016) auditorily presented DRM lists. The participant heard each word once with a 1 s delay between consecutive words. We selected the lists from a study by Stadler et al. (1999) who evaluated 36 lists for their level of identifiability. To determine if identifiability (i.e., how easily a person can detect the critical lure) was similar across phases of the study, we evaluated the backward association strength (BAS; how strongly associated the critical lure is to the list words) using normed mean BAS scores from Roediger et al. (2001). The average BAS scores (phase 1 studied words = 0.216, phase 2 studied words = 0.167, and novel non-studied words = 0.178) are fairly consistent across conditions, indicating a similar level of free recall association between the critical lure words and the list words.

In the first phase of the experiment, participants heard eight DRM lists in a randomized order, each containing fifteen words presented in the standard manner of descending order of association to the non-presented critical lure. Examples of the lists are shown in Table 1.

**TABLE 1:** Sample DRM lists with critical lures typed in bold and underlined.

Anger	Chair	Doctor	Mountain
Mad	Table	Nurse	Hill
Fear	Sit	Sick	Valley
Hate	Legs	Lawyer	Climb
Rage	Seat	Medicine	Summit
Temper	Couch	Health	Top
Fury	Desk	Hospital	Molehill
Ire	Recliner	Dentist	Peak
Wrath	Sofa	Physician	Plain
Happy	Wood	Ill	Glacier
Fight	Cushion	Patient	Goat
Hatred	Swivel	Office	Bike
Mean	Stool	Stethoscope	Climber
Calm	Sitting	Surgeon	Range
Emotion	Rocking	Clinic	Steep
Enrage	Bench	Cure	Ski

*Lists are from Roediger and McDermott (1995).*

After hearing all eight lists, the participants performed a visual recognition task in which they had to decide if a test item was an old word (i.e., presented on the previous lists) or a new word (i.e., had not been heard previously). The recognition task consisted of 56 trials which included 24 words from the DRM lists (3 from each list; positions 1, 5, and 11), the critical lure words from all eight lists, and 24 words that were novel with low association with the DRM lists. Participants saw each word one at a time with options, labeled on the screen, to press “Z” to indicate the word was old and “/” to indicate the word was new. Words appeared on a white background in black print in the center of the screen. Each word remained onscreen until selection, and we recorded the decision time. Following the selection, a blank screen appeared for 5 s so that we could continue to measure the hemodynamic response. Next, participants saw a confidence rating scale which asked them to rate their level of confidence for each of their old/new choices. The scale appeared in increments of five and ranged from *not at all confident* (0) to *very confident* (100). We averaged each trial confidence score across all forms of correct and incorrect recognition. The next trial began 500 ms later.

After the first phase was complete, we explained the nature of DRM lists and their corresponding critical lures to participants. Next, in the 15-item word lists but before each, we informed them of the critical lure word that was not on the list. They were instructed to recognize the critical lure as an old word on the memory test during the deception phase, despite knowing it was not on the list. Seven trials immediately followed each of the eight lists and included three words from the list, three novel words, and the critical lure. Participants again had to determine if these words were “old” or “new” by selecting the “Z’ or “/” key and then rate their confidence on a 0–100 scale.

Finally, participants answered a brief demographic questionnaire and completed an 18-question Need for Cognition scale (Cacioppo and Petty, 1982). Individuals who have a higher need for cognition tend to commit more false memories than individuals with a lower need for cognition (Graham, 2007; Leding, 2011).

Before calculating oxyhemoglobin levels, we preprocessed each participant’s raw optical data. To do this, we used NIRx nirsLAB software (version 2019.04, NIRx Medical Technologies, LLC, Brooklyn, NY, United States). To check the data’s signal-to-noise ratio, we used a coefficient of variance filter of 7.5%. We considered any channels that exceeded this threshold as noisy and excluded them from the data. We used a band pass filter (0.01–0.2 Hz) to filter the data and capture the effects of our experimental paradigm. This reduced the effects of physiological noise, such as heart and respiration rates.

Finally, we used the modified Beer-Lambert Law to calculate oxyhemoglobin levels. The refraction path of infrared light is affected by its travel through bone and tissue, and the thickness and density of these differs with age. Accordingly, we used each participant’s age to set the differential path link factor parameter for each wavelength of light, as suggested by Scholkmann and Wolf (2013). This is important because we used the differential path link parameter to calculate oxyhemoglobin levels. We block averaged the resulting oxyhemoglobin levels across memory conditions for each channel for each participant and exported them from nirsLAB to SPSS for statistical analysis. (NIRScout also measures deoxygenated and total hemoglobin levels, but as most of the current fNIRS literature primarily focuses on less-noisy oxyhemoglobin levels, we have done the same). For each phase of the study, we compared cortical activity that occurred 1 s before (as a baseline) to the maximum oxyhemoglobin response occurring within 5 s after each event marker. Thus, for the first and deception phases of the experiment, we examined oxyhemoglobin levels within 5 s after participants were asked to indicate whether a given word was old or new. According to Vega et al. (2016), the hemodynamic response to a participant’s lie or truth occurs during this timeframe.

Because most of our participants had a few noisy channels that we removed from the data, we used the SPSS mixed procedure to estimate a two-factor (6 memory conditions × 6 brain regions) within-subjects ANOVA (Enders, 2010). The memory conditions were Correct Old Memory (correct recollection of words on the DRM lists), Correct New Memory (correct rejection that a new word was not on the lists), Wrong Old (incorrectly stating that a word was not on the list when it was), Wrong New (incorrectly stating that a novel word was on the list when it was not), False Memory (incorrectly stating that a lure word was on a list when it was a new word), and Deception (participant stated the critical lure was on a DRM list after being instructed to do so when it was a new word). We assessed three brain regions of interest separately in the left and right frontal lobes: two channels in each anterior prefrontal cortex (APFC), eight channels in each dorsolateral prefrontal cortex (DLPFC), and five channels in each ventrolateral prefrontal cortex (VLPFC). We measured the peak oxyhemoglobin response for each channel during the 5-s period following each response and, for each region of interest, averaged their channels’ peak responses. Data for Correct New Memories, Correct Old Memories, Wrong New Memories, Wrong Old Memories, and False Memories were each pooled across the recognition and deception phases. Paired-sample *t*-tests assessed specific differences in memory conditions in each region of interest. To correct for multiple pairwise comparisons, we excluded those that did not meet a threshold of *p* ≤ 0.01.

### Results

#### Behavioral Factors

We evaluated and compared reaction time, confidence, false memory, true memory, and correct rejection rates, as well as need for cognition. Average response time across participants was 7.33 s (*SD* = 0.38) during the course of the experiment. All participants had at least one false memory, with an average of 6 (79.74%). Participants on average correctly responded to 16.69 studied words, true memory (69.54%), and 19.24 non-studied, novel words by selecting “new” (80.17%; thus, only about a 20% false recognition rate for novel items). Participants on average correctly responded to 6.21 deception words (77.65%). The frequency of false memories was positively correlated with the number of true memories [*r*(29) = 0.55, *p* = 0.002]. Rates of false memories of critical lures and misremembering novel words were consistent with previous literature (Toglia et al., 1999; Prohaska et al., 2016).

Participants’ average confidence on responses was 66.92 (*SD* = 13.11). A paired samples t-test revealed no significant difference between true memory confidence (*M* = 68.55, *SD* = 13.63) and false memory confidence (*M* = 73.46, *SD* = 15.66). The number of false memories (*M* = 6.37, *SD* = 1.84) was positively correlated with confidence [*r*(31) = 0.49, *p* < 0.001] while we found no relationship between the number of true memory or correct rejections and confidence. Additionally, the number of novel words reported as old (*M* = 5.61, *SD* = 4.98) was positively correlated to confidence as well [*r*(31) = 0.44, *p* = 0.019]. Notice that accuracy was not associated with confidence, while inaccuracy was positively related to confidence consistent with the general conclusion in the literature that confidence in memory does not guarantee its accuracy.

The number of false memories was negatively related to false memory reaction time (*M* = 7.58, *SD* = 0.77), meaning that the more false memories a participant had, the faster their response when committing a false memory of critical lures [*r*(31) = −0.39, *p* = 0.02]. This pattern did not occur for false memories of novel words. We found no difference between response time for correct rejections or true memories compared to false memory responses but participants took significantly longer to respond when committing a false memory compared to when telling an intentional lie (*M* = 7.01 ms, *SD* = 0.85), [*t*(29) = −3.77, *p* < 0.001].

#### Need for Cognition

We evaluated Need for Cognition (Cacioppo and Petty, 1982) by using a mid-point of zero: a positive score indicated a high need for cognition, a negative score indicated a low need for cognition. On the Need for Cognition scale, which ranged from −72 to 72, participants averaged 24.34. Higher scores were positively correlated with confidence in correct responses to studied words [*r*(29) = 0.34, *p* < 0.05], incorrect responses for studied words [*r*(29f) = 0.39, *p* < 0.05], and correct responses to non-studied words [*r*(29) = 0.52, *p* < 0.005]. Need for Cognition was also positively correlated with the number of correct responses to studied words [*r*(29) = 0.37, *p* < 0.05] but was not related to false memory rate, reaction time, nor false memory confidence.

##### Brain Activation

A repeated measures ANOVA found main effects for brain region [*F*(5,3278) = 6.03, *p* < 0.001, *f* = 0.088] and memory condition [*F*(5,3265) = 15.16, *p* < 0.001, *f* = 0.147] and no omnibus interaction between brain region and memory conditions [*F*(5,3264) = 0.92, *p* = 0.58]. The significant pairwise comparisons (*p* ≤ 0.01) for brain regions and memory conditions appear in Table 2.

**TABLE 2:** Pairwise comparisons for memory conditions and brain regions of interest.

(I) Memory	(J) Memory	Mean difference (I–J)	df	Sig
Correct new (Correct rejection)	Correct old (Hit)	2.79E-06	3264.113	0.851
	Deception	−7.431E-5^*^	3267.423	<0.001
	False	−7.080E-5^*^	3263.795	<0.001
	Wrong new	−9.522E-5^*^	3263.795	<0.001
	Wrong old (miss)	−3.997E-5^*^	3263.795	0.007
Correct old (hit)	Deception	−7.710E-5^*^	3267.839	<0.001
	False	−7.359E-5^*^	3264.113	<0.001
	Wrong new	−9.801E-5^*^	3264.113	<0.001
	Wrong old	−4.276E-5^*^	3264.113	0.004
Deception	False	3.51E-06	3267.423	0.814
	Wrong new	−2.09E-05	3267.423	0.162
	Wrong old	3.434E-5	3267.423	0.022
False	Wrong new	−2.44E-05	3263.795	0.099
	Wrong old	3.083E-5	3263.795	0.037
Wrong new (false alarm)	Wrong old	5.525E-5^*^	3263.795	<0.001

**(I) ROI**	**(J) ROI**	**Mean difference (I–J)**	**df**	**Sig**

LDLPFC	LAPFC	5.096E-5^*^	3278.775	<0.001
	LVLPFC	3.287E-5	3273.316	0.026
	RDLPFC	1.21E-05	3263.956	0.401
	RAPFC	7.151E-5^*^	3278.457	<0.001
	RVLPFC	3.837E-5^*^	3273.355	0.009
LAPFC	LVLPFC	−1.81E-05	3289.922	0.235
	RDLPFC	−3.884E-5^*^	3279.203	0.009
	RAPFC	2.06E-05	3271.672	0.178
	RVLPFC	−1.26E-05	3288.178	0.409
LVLPFC	RDLPFC	−2.08E-05	3271.615	0.159
	RAPFC	3.864E-5^*^	3289.577	0.011
	RVLPFC	5.50E-06	3271.86	0.714
RDLPFC	RAPFC	5.939E-5^*^	3278.886	<0.001
	RVLPFC	2.63E-05	3271.67	0.075
RAPFC	RVLPFC	−3.314E-5	3287.841	0.029

LDLPFC, left dorsolateral prefrontal cortex; LAPFC, left anterior prefrontal cortex; LVLPFC, left ventrolateral prefrontal cortex; RDLPFC, right dorsolateral prefrontal cortex; RAPFC, right anterior prefrontal cortex; RVLPFC, right ventrolateral prefrontal cortex. * indicates (p ≤ 0.01).

True memories (correctly remembering that a word was on a DRM list) produced significantly less activation of prefrontal areas than did Deception, False Memories, Wrong Old memories, and Wrong New memories. Also, incorrectly stating that a new word was on a list (Wrong New) produced more activation than failure to recall that a word was on a list (Wrong Old).

During all our memory conditions, we found significantly more activation in the left dorsolateral prefrontal cortex than the right VLPFC and both left and right APFC. In addition, we measured greater activity in the right DLPFC than both left and right APFC, and greater activity in the left VLPFC compared to right APFC.

Despite lack of a global interaction, pairwise comparisons indicated several significant differences (*p* ≤ 0.01) between memory conditions in specific prefrontal regions. Table 3 presents these comparisons.

**TABLE 3:** Significant differences (*p* ≤ 0.01) between memory conditions in specific prefrontal regions.

Brain region/memory condition	Brain region/memory condition	Mean difference	*t*	df	Sig
LDLPFC correct old (hit)	RDLPFC correct old	2.97E-05	2.721	27	0.011
	LAPFC correct old	4.48E-05	2.801	25	0.010
	RAPFC correct old	5.03E-05	3.740	25	0.001
LDLPFC correct new (correct rejection)	RVLPFC correct new	5.80E-05	3.519	26	0.002
	LAPFC correct new	4.08E-05	3.712	25	0.001
	RAPFC correct new	5.89E-05	5.106	25	0.000
RDLPFC correct new (correct rejection)	RAPFC correct new	4.56E-05	3.499	25	0.002
LDLPFC correct lure (correctly reject lure)	LDLPFC correct old	2.05E-04	2.690	21	0.014
RDLPFC correct lure	RDLPFC correct old	2.25E-04	3.223	20	0.004
	RDLPFC correct new	1.81E-04	2.869	21	0.009
LDLPFC wrong old (miss)	LVLPFC wrong old	8.91E-05	3.709	26	0.001
	LAPFC wrong old	9.40E-05	3.590	25	0.001
	RAPFC wrong old	5.26E-05	2.979	25	0.006
RDLPFC wrong old (miss)	LVLPFC wrong old	1.01E-04	3.792	26	0.001
	LAPFC wrong old	1.07E-04	4.832	25	0.000
	RAPFC wrong old	6.80E-05	2.688	25	0.013
	RDLPFC correct old	1.07E-04	3.697	27	0.001
	RDLPFC correct new	7.97E-05	3.186	28	0.004
LDLPFC wrong new (false alarm)	LDLPFC correct old	1.22E-04	3.025	28	0.005
	RAPFC wrong new	1.18E-04	3.035	25	0.006
RDLPFC wrong new	RDLPFC correct old	1.35E-04	3.568	27	0.001
	RDLPFC correct new	1.05E-04	3.046	28	0.005
	RAPFC wrong new	1.03E-04	3.415	25	0.002
LVLPFC wrong new	LVLPFC correct old	1.11E-04	2.715	26	0.012
	LVLPFC wrong old	1.27E-04	3.220	26	0.003
	LVLPFC correct new	1.14E-04	2.685	26	0.012
RVLPFC wrong new	RVLPFC correct new	9.56E-05	2.663	26	0.013
RAPFC wrong new	RAPFC correct new	6.61E-05	2.702	25	0.012
LDLPFC false memory	LDLPFC correct old	6.15E-05	2.652	28	0.013
	RAPFC false memory	6.81E-05	3.584	25	0.001
RDLPFC false memory	RDLPFC correct old	7.45E-05	2.717	27	0.011
	RAPFC false memory	4.85E-05	3.187	25	0.004
LVLPFC false memory	LVLPFC wrong old	8.97E-05	2.666	26	0.013
LAPFC false memory	LAPFC correct old	1.30E-04	3.540	25	0.002
	LAPFC wrong old	1.15E-04	3.207	25	0.004
	LAPFC correct new	1.26E-04	3.947	25	0.001
	RAPFC false memory	9.27E-05	3.107	24	0.005
LDLPFC deception	LDLPFC correct old	7.21E-05	3.069	27	0.005
	RAPFC deception	6.46E-05	3.303	25	0.003
RDLPFC deception	RDLPFC correct old	1.01E-04	2.902	26	0.007

*LDLPFC, left dorsolateral prefrontal cortex; LAPFC, left anterior prefrontal cortex; LVLPFC, left ventrolateral prefrontal cortex; RDLPFC, right dorsolateral prefrontal cortex; RAPFC, right anterior prefrontal cortex; RVLPFC, right ventrolateral prefrontal cortex.*

Correct Old memories (“hits” – correct recognition of words presented on the DRM lists) induced greater activation in the left DLPFC than the left APFC, right APFC, right DLPFC, and right VLPFC. Correct Old memories (hits) also produced higher activation in the right DLPFC than right APFC. In both left and right DLPFC, being correct about a critical lure produced more activity than did true memory hits.

Compared to Correct Old memory hits, incorrect memories (Wrong Old “misses” or Wrong New “false alarms”) led to increased activity in left DLPFC, left VLPFC, right DLPFC, right VLPFC, and right APFC. This was especially notable in the right DLPFC where each incorrect memory condition produced greater activity than true memory hits. Wrong Old memories (misses) produced the greatest activity in left and right DLPFC while a pattern was less clear for Wrong New memories (false alarms).

Compared to true memory hits, participants’ False memories led to greater activation in the left DLPFC, the right DLPFC, and the left APFC, and False memories produced greater activity in those three regions than they did in right APFC. Compared to Wrong Old memories (misses; forgetting an old word), false memories induced greater activation in the left APFC and the left VLPFC. Similar to False memories, Deception produced more activation in the left DLPFC and the right DLPFC when compared to hits. Deception produced higher levels of activity in the left DLPFC than right APFC.

We used bivariate correlations to compare confidence scores to oxygenated hemoglobin levels for each condition within each region of interest. Confidence levels were positively correlated with hemoglobin levels in the right VLPFC when participants incorrectly stated that new words were old [Wrong New memories; false alarm, *r*(29) = 0.46, *p* = 0.02] and old words were new [Wrong Old memories; miss, *r*(29) = 0.61, *p* = 0.001]. For forgotten old words (Wrong Old memories; misses), confidence increased with oxygenated hemoglobin in the left VLPFC [*r*(29) = 0.38, *p* = 0.05]. Finally, in the left DLPFC, confidence increased with oxygenated hemoglobin in the False Memory (critical lure) condition [*r*(29) = 0.38, *p* = 0.04].

### Discussion

#### True and False Memories

False memory data for critical lures yielded significant behavioral effects and brain activation. Higher rates of false memories correlated with true memories. This pattern is consistent with previous research showing that better recall of list items is associated with more false memories and that this relationship is stronger when word lists are thematically blocked as opposed to randomly ordered (Toglia et al., 1999). According to Fuzzy Trace Theory, a gist understanding of the word lists helps participants recognize previously presented words, but also increases the likelihood of false memories. This pattern is also consistent with Activation-Monitoring Theory which predicts that target words and critical lures are highly and repeatedly activated at encoding and pass a monitoring examination during retrieval. Individuals with better accurate (true) memory and more false memories might pay closer attention to the list presentations, and they might use strategies such as chunking which could make them more likely to misremember semantically related words. We found that participants with higher need for cognition had better recall, further supporting this idea. On the other hand, our participants with high need for cognition did not have more false memories, contrary to prior research (Graham, 2007; Leding, 2011). Those high in need for cognition did, however, have more overall confidence in their ratings regardless of the number of false memories (Kuvaas and Kaufmann, 2004).

Participants with more false memories of critical lures responded faster and were more confident in their responses. The quicker reaction time might indicate more reliance on gist trace memories rather than verbatim memory. Interestingly, response time was not related to novel false memories despite having a similar correlation with confidence. Fuzzy Trace Theory suggests that inhibitory processes repress false memory responses (Reyna and Mills, 2007), thus the faster response time might indicate that these participants are not engaging in as much inhibitory control. Our fNIRS data, however, which indicates more activity in the prefrontal cortex during false memories compared to true memories, does not fit that interpretation. The forensic psychology literature has seen a push to distinguish accuracy from confidence (Busey et al., 2000; Chua et al., 2004; Storbeck and Clore, 2005). Often in the legal setting jurors assume that a person who is confident in their testimony must be correct, but many studies have shown little to no correlation between these constructs, a premise our study supports (Sporer et al., 1995; Ais et al., 2016). Together these results suggest that individuals are more confident despite having greater levels of false memories, which then might lead to them respond more quickly. Thus, neither confidence nor reaction time serve as proxies for accuracy. High confidence scores during false memories fall in line with AMT and the spreading activation theory. As participants encode words they are likely creating associative networks that include the critical lures. This would make it more likely for participants to perceive these lures as target words and do so confidently.

Several areas of the prefrontal cortex were activated during all of our memory conditions but false memories (falsely remembering a critical lure) and, similarly, incorrectly thinking that other new words were old, produced more activity in the prefrontal cortex than did true memories and correct rejections. The prefrontal region most activated by true memories was the left DLPFC while false memories increased hemoglobin levels in both left and right DLPFC. Yu et al. (2019) performed a meta-analysis of 77 fMRI studies and similarly concluded that while false memories recruit several regions of the frontal, parietal, and temporal lobes, the left DLPFC is more activated by false memories than true ones. The DLPFC, especially on the right side, according to some researchers, has been implicated in the appraisal of the value of the memory for current task performance during post-retrieval monitoring (Henson et al., 1999; Achim and Lepage, 2005; Chua et al., 2009; Wang et al., 2016). Increased DLPFC activity might also reflect the monitoring component of AMT as participants evaluate whether a lure was on the list. This would require more cognitive effort and likely some inhibition if their first instinct is to semantically associate the lure word. We also found an increase in activity in the left APFC. Using positron emission tomography (PET) with the DRM paradigm, Schacter et al. (1996) found a similar pattern of activation, and, by measuring increased blood flow in the APFC and PFC, they were able to distinguish false from true memories.

Kurkela and Dennis (2016) meta-analysis also implicated multiple prefrontal regions, mostly medial to those accessible to fNIRS, as well as the bilateral inferior frontal gyri which corresponds to the VLPFC where we found, in the left hemisphere, that false memories elicited more activity than forgetting an old word did. This was unexpected because previous research has implicated the right, not left, VLPFC in evaluating uncertain information for accuracy (Chua et al., 2009; Goel et al., 2009; Ito et al., 2012). Further research is required to untangle this discrepancy.

Because true and false memories tend to mostly activate the same cortical regions (Johnson et al., 1997; Schacter and Addis, 2007; Mitchell and Johnson, 2009; Kurkela and Dennis, 2016; Yu et al., 2019), it is difficult to determine whether there are definitive gist neural pathways or regions that respond specifically to false memory information. Our results are consistent with previous literature and are congruent with both FTT and AMT, but larger imaging studies focusing on areas of distinction such as the anterior prefrontal cortex, are needed to distinguish any subtle differences between these responses.

#### Deception

Numerous imaging and brain stimulation studies have confirmed that the prefrontal cortex is the generator of lies, whether the lie is simply a response to an instruction by an experimenter or is a spontaneous and deliberate attempt to deceive. Lying involves several cognitive processes that are thought, at least in laboratory situations, to require more mental effort than does telling the truth. A liar must hold remembered information in working memory, consider the consequences of lying vs. truth-telling, suppress the urge to voice the truth, concoct a narrative that is contrary to the truth but still believable, attempt to avoid “tells” by regulating eye movements, facial expressions, and body language, and infer how the lie is being received by the listener (for review see Gombos, 2006).

In our DRM study, compared to true memories, deception produced more activation in prefrontal areas, especially the left and right DLPFC. This conforms with a large meta-analysis of fMRI studies which also found that deception, whether instructed or spontaneous, increased PFC activity, especially in the left and right DLPFC (Yu et al., 2019). fNIRS has yielded similar results (Ding et al., 2013, 2014) and very recently Lin et al. (2021) used fNIRS to measure cortical activity in the DLPFC and the APFC while participants lied while playing a poker game with an opponent. Both prefrontal areas were more active when participants lied versus when they told the truth. Interestingly, Li et al. (2018) used fNIRS to show that the left middle frontal gyrus, which overlaps with the left DLPFC, reacts most strongly to deceptive responses by participants who lie rarely compared to those who lie regularly or to those who tell the truth.

It is notable that all of our memory conditions increased hemoglobin levels in the left DLPFC. Ito et al. (2012) suggest that creating all types of memory and lies are taxing and that the left DLPFC is responsible for preparing us for both truthful and deceptive responses. They used fMRI to measure brain activity while participants prepared to tell the truth or to lie about photographs they had been shown, and then, several seconds later, during the actual lie or truth-telling. The left DLPFC was significantly more active during the preparation phase when participants knew whether they would be asked to tell the truth or to lie compared to trials in which they did not know in advance whether they would be asked to respond with truth or deception. Interestingly, during this preparation phase, the left DLPFC was equally active in both the truth and lie conditions. During the execution phase, however, they found, as we did, that the left DLPFC was more active when participants told a lie than when they told the truth.

These results support cognitive load theories of deception which suggest that executing a lie requires more cognitive resources than truth telling because telling the truth is usually an automatic response while lying involves additional steps such as constructing an alternative response while suppressing possible indicators of deception. These extra steps require the recruitment of additional cognitive resources while the lie is in progress (Zuckerman et al., 1981; Vrij et al., 1996; Walczyk et al., 2003). It is possible that in our experiment, cognitive load increased further when we asked participants to report that the critical lure was a lie. According to FTT, remembering specific words, as opposed to the lists’ themes, requires reliance on verbatim trace memory and is likely to require more cognitive effort than simply remembering similar words with a semantic association. This increased cognitive load is probably also reflected in the DLPFC when we asked our participants to lie.

Further increasing cognitive load by adding additional effortful tasks can expose lies. Vrij et al. (2008) required mock suspects to tell their story to police officers in a chronologically backward order. With this additional cognitive load, the suspects could not suppress noticeable deception cues and the officers were better able to detect the liars. Our results, as well as those of others, show that fNIRS can detect differences in cognitive load (Fishburn et al., 2014) and could be used as an inexpensive, portable, and effective lie detector, at least for infrequent liars who seem to experience less cognitive load during deception (Li et al., 2018). Using fNIRS, computers and humans can be trained to distinguish truth from lies by simply viewing static images that reflect the relative changes in hemoglobin levels occurring in an interviewee’s prefrontal cortex during questioning (Vega et al., 2016). Combining fNIRS with a traditional polygraph system is an even better solution that significantly improves lie detection (Bhutta et al., 2015).

Though both false memories and deception produced more activation in prefrontal areas than true memories and correct rejections, we did not find an activation difference between the two conditions. This may indicate that our low-stakes deception task, in which participants were instructed to lie about remembering words on a list, does not produce the same level of cognitive processing required of intentional face-to-face deception. Indeed, Lin et al. (2021) report that in their poker game study, deliberate spontaneous deception produces higher activity in both the DLPFC and APFC than when participants are merely told to lie. In a similar recent fNIRS study with card players that focused exclusively on the APFC, the highest levels of activity occurred only in intentional face-to-face deception (Pinti et al., 2021). This most anterior region of the prefrontal cortex, which is poorly understood, has been implicated in numerous cognitive tasks including episodic memory retrieval, social cognition, and mentalizing – all tasks that are presumably necessary to meet the demands for effective intentional deception (Gilbert et al., 2006; Burgess et al., 2007). Taken together, our findings suggest that all types of memory activate the prefrontal cortex, especially the DLPFC, but false memories and deception, which likely require more cognitive resources, result in even higher levels of activity and that the coordination of several regions of the prefrontal cortex is involved in these processes.

### General Discussion

Twenty-five years of research shows that the DRM (Deese–Roediger–McDermott) paradigm impressively produces false memories of non-presented critical lures as well as inaccurate remembering of other lures. These compelling indications of the DRM illusion occurred in the two investigations we report in this article. False memory researchers continue to publish new demonstrations of the DRM illusion while concentrating on advances that fall into at least three categories: theoretical accounts, strategies for reducing false memories, and real-world implications. As we address these classifications, we begin by briefly mentioning the disparate nature of our LDT and fNIRS investigations and then turn to spelling out the relationships between these research agendas.

They are certainly different lines of research. Study1 introduces a new approach to engendering false memory by using a lexical decision task as a priming agent. The fNIRS (functional near-infrared spectroscopy) experiment measures oxygenation changes in the prefrontal cortex. Our purpose was to leverage cognitive neuroscience findings to guide analyses of cortical changes across truth-telling, deception, and false memory. In a mostly traditional DRM paradigm, we examined these three in a within-participants design, a combination that is not a common research strategy (again see Abe et al., 2008 for a similar study). Deception occurred *via* intentional lying instructions, and in all phases of this experiment we collected neural correlate data. These different approaches to studying illusory memory share many similarities which permit an interesting window into how results from one DRM priming technique (LDT) under incidental memory testing conditions generalize to findings obtained under another DRM experimental design using intentional memory instructions. Indeed, the behavioral results for true and false memory across experiments indicate DRM priming is powerful even without intent to remember.

Response times were similar for true and false memories, a finding observed in both the laboratory project and the fNIRS study, so RT was not a distinguishing factor. Both investigations tested recognition memory with a focus on DRM-created memories in the genre of spontaneous false memories, as opposed to implanted (suggested) false remembrances. In DRM studies, including our LDT and fNIRS research, experimenters do not actively cause inaccuracies. Rather, *via* autosuggestion participants generate faulty theme-consistent memories (Brainerd and Reyna, 2005). These semantically based illusory memories are consistent with AMT’s positions concerning activation at encoding and monitoring failures at retrieval, and with FTT’s reasoning grounded in the formation of strong gist traces. These theories are compatible with the more-is-less pattern of increases in true memory accompanied by increases in false memory (Toglia et al., 1999) that we saw in both investigations. Though participants demonstrated very good target recognition, their overall accuracy suffered by committing high levels of false recognition memory. This constitutes an argument for memory impairment in the DRM paradigm.

Both investigations employed many of the same DRM lists. The LTD studies involve the lists of Toglia et al. (1999) which successfully fomented false memories at levels sufficient to potentially see the illusion with incidental memory assessment. In the fNIRS experiment the chosen DRM study lists corresponded to highly “identifiable” critical lures. This means that the LDT lists also referred to decidedly identifiable critical lures. Regardless, robust false memory levels occurred in the present investigations! This suggests that in daily life some false information is likely discernable as untrue and yet people may believe such easy to identify false messages, subsequently expressing them as memories deemed accurate. Thus, identifiability does not seem to serve as an implicit warning to reduce false memory, a conclusion consistent with failures of explicit warnings to attenuate illusory recollections (Neuschatz et al., 2001). Our behavioral findings on lying are relevant here because instructions to intentionally lie are the ultimate form of warning participants of the existence of the DRM illusion. Nevertheless, we saw that participants failed about 20% of the time to provide the lie that critical lures had been previously heard. Apparently, they ignored or forgot the instructions. Forgetting is quite possible because we’d expect verbatim traces of instructions to fade quickly in this context (Reyna and Brainerd, 1995). A related possibility involving loss of verbatim information, raises the notion that a memory lapse here is more complex than just not recalling an instruction. What if participants simply do not remember some of the critical lures for which they were asked to provide a lie? This scenario or a combination of the two suggest forgetting is far more plausible than mere ignorance. Thus, lying might not always occur under conditions that should permit perfect identifiability of the lure words. We also favor this forgetting explanation because it fits with the cognitive load problems exposed in the fNIRS study.

The preceding argument leads to two issues. The first is a reminder of links between false memory and deception which portend real-world applications. Our fNIRS findings, which show different degrees and/or patterns of activation in several prefrontal regions for true memories, false memories, and lies, are consistent with the notion that these constructs are in fact different (Ceci et al., 1987; Loftus, 2005).

The second, as we mentioned, is that similarities between false memory and deception points to a positive example of ecological validity. We are aware of concerns about the external validity of the DRM procedure (Baioui et al., 2012), but the paradigm offers a straightforward way to examine issues relevant to a large body of work on schematic knowledge. Also, as noted in the Introduction, DRM experiments yield findings that line up with experimental results reported with stimulus materials regarded as more ecologically valid. These stimuli include sentences, prose passages, pictures, and visual scenes.

External validity leads us to thoughts on future paths for behaviorally-oriented DRM research and for the paradigm’s role in advancing strides in cognitive neuroscience. As in many DRM studies, our participants are young adults. Extending both of our investigative techniques to testing children, middle-aged adults, and older adults will diversify our sample and add to the literature concerning the developmental trajectory of false memories (Brainerd et al., 2011). Our methodologies, especially the LDT priming, might also be useful for special populations – like individuals with intellectual disabilities such as autism spectrum disorder (Beversdorf et al., 2000). Our LDT procedure would enable us to explore individual differences such as the Need for Cognition trait that we included in the fNIRS study. Our recommendations to this juncture have been at the behavioral level.

At the neurophysiological level, considerable brain imaging research has attempted to distinguish between true and false memories and we have cited a number of studies that converge on the conclusion that these memories generally tend to show activation in the same cortical areas. Our current experiments are concordant with this conclusion but a nagging question is whether thematically-oriented pathways exist that signal the presence of false messages and lies. Answering this will require more extensive imaging experiments that concentrate on regions of distinction such as the anterior prefrontal cortex.

## Data Availability Statement

The original contributions presented in the study are publicly available. This data can be found here: fNIRs PFC Memory and Deception, https://www.nitrc.org/projects/fnirs_drm_2021/.

## Ethics Statement

The studies involving human participants were reviewed and approved by Office of Research & Sponsored Programs, University of North Florida, Jacksonville, FL 32224. The patients/participants provided their written informed consent to participate in this study.

## Author Contributions

MT organized and wrote the manuscript. JS developed the new paradigm which is the basis of Study 1 and with MT conducted a modified follow-up experiment, wrote much of the prose for Study 1, and proofed the introductory section written by MT. BS leveraged her master’s thesis advised by MT to become Study 2 in this article, conducted additional analyses, and proofed this section. KH wrote and proofed with BS the Study 2 section, edited the general discussion written by MT, and gathered the datasets in setting up the availability repository. ND worked on Study 1, proofed several sections of the present manuscript, set up the initial version of this template, and prepared the final versions of our figures. BW collected the data in the follow-up experiment within Study 1, assisted BS when she collected additional data beyond her thesis, and edited the manuscript. All authors contributed to the article and approved the submitted version.

## Conflict of Interest

The authors declare that the research was conducted in the absence of any commercial or financial relationships that could be construed as a potential conflict of interest.

## Publisher’s Note

All claims expressed in this article are solely those of the authors and do not necessarily represent those of their affiliated organizations, or those of the publisher, the editors and the reviewers. Any product that may be evaluated in this article, or claim that may be made by its manufacturer, is not guaranteed or endorsed by the publisher.
